# Nurses’ perspectives on workplace environment needs associated to resilience: a qualitative descriptive study

**DOI:** 10.3389/fpsyt.2024.1345713

**Published:** 2024-02-09

**Authors:** Meng Li, Runze Zhao, Junfan Wei, Linghan Zhou, Shuhua Yang, Yuan Tian, Lingning Wang, Wenling Zhang, Xiaoyun Xiong, Chuzhen Huang, Zhongjie Pan, Ruipeng Song

**Affiliations:** ^1^ Nursing Department, The Third People’s Hospital of Henan Province, Zhengzhou, China; ^2^ School of Medicine, Zhengzhou University of Industrial Technology, Zhengzhou, China; ^3^ The Seventh Clinical Medicine College of Guangzhou University of Chinese Medicine, Shenzhen, China; ^4^ School of Medicine, Maanshan University, Maanshan, China; ^5^ Operating Room, Henan Provincial People’s Hospital, Zhengzhou, China

**Keywords:** nurses, psychological resilience, workplace wellbeing, cognition, experience nurses, experience

## Abstract

**Objective:**

The purpose of this study was to explore the demands of nurses on the workplace environment related to psychological resilience.

**Methods:**

A qualitative descriptive design was employed for this study. Purposeful sampling was chosen from a tertiary hospital in Henan Province, China. Semi-structured in-depth interviews were conducted with 20 nurses. The interview data was analyzed using the Colaizzi’s method and results were reported following the COREQ standards.

**Results:**

Analysis of the interview data revealed three main themes: (1) Career Support and Development, (2) Practical Support & Development, and (3) Personal Support and Development.

**Conclusion:**

The perspectives of nurses for a workplace environment demands needs to be appreciated, and in addition, it is worth noting that the key role of building a good workplace environment in strengthening the resilience of nurses emphasizes the need for careful consideration. Nursing administrators should formulate policies and measures from multiple perspectives based on the real needs of nurses in terms of professional, practical, and personal dimensions.

## 1 Introduction

Issues such as immediate patient care complexity, administrative mandates, the need to constantly improve professional skills, and dealing with occupational hazards and potential errors in high-stress settings are numerous (1). Such intrinsic challenges, while integral to the nursing profession, can negatively impact their psychological state, leading to outcomes such as emotional fatigue, eroded self-esteem, heightened stress, and the looming threat of burnout (2). Such repercussions contribute to the burgeoning issue of nurse attrition, further accentuating the global nursing shortage dilemma, potentially compromising the quality of patient care and obstructing the broader advancement of the nursing field (3).

Psychological resilience (4), characterized as an individual’s adaptive prowess in the face of adversities and setbacks, plays a pivotal role in how professionals, especially nurses, cope in their demanding roles. An enhanced psychological resilience framework could act as a bulwark against the detrimental effects of emotional labor in healthcare settings (5).

Nurse workplace environment is an important subsystem of hospital environment system, which is the product of hospital nursing management, nursing human resource allocation, work design and organizational culture. Nurse workplace environment is related to the physical and psychological well-being, and quality of work life (QWL) for nurses (6). Another survey study of nurse directors indicated that the workplace environment had an impact on nurses’ job burnout (7).

At the same time, psychological resilience has a complex relationship with nurses’ workplace environment. One survey (8) study found that improvements in the workplace environment can have a positive impact on mental health outcomes. The Health Service Workplace Environmental Resilience Model (HSWERM), which introduced by Cusack (9) et al. in 2016, emphasizes the components of the nursing workplace environment that interplay with psychological resilience. Although too much qualitative research has focused on nurses’ adverse experiences, emotional states, life narratives and challenges, there is still a lack of research on the impact of workplace environment on resilience (10). Limited research has ventured into capturing the authentic sentiments and lived experiences of nurses concerning their support necessities in a resilience-conducive practice setting.

The Health Service Workplace Environmental Resilience Model (HSWERM), introduced by Cusack (9) et al. in 2016, emphasizes the components of the nursing workplace environment that interplay with psychological resilience. This intricate model threads through dual dimensions — namely, support and development — and operates on three planes: professional, practical, and personal. Based on this conceptual framework, we conducted an in-depth dialogue with nurses in the survey, aiming to reveal their internal needs for the workplace environment related to psychological resilience. The results of this study provide a reference guide and fundamental impetus to provide new intervention ideas for the improvement of psychological resilience of nurses, and guide related research to an unparalleled refinement.

## 2 Research subjects and methods

### 2.1 Participants

We employed a face-to-face interview approach,; each interview was conducted individually. The participants were selected via convenience and purposive sampling and were drawn from a tertiary care hospital in Henan Province. The inclusion criteria for nurses were: 1) Currently employed; 2) Engaged in clinical nursing work; 3)Having more than 1 year of work experience; 4) Informed and voluntarily agreed to participate. We excluded participants who were: 1) Trainees or interns; 2) Not working in the hospital during the survey period, including those out for further study, on sick leave, personal leave, or maternity leave. The sample size was determined by data saturation. Finally, a total of 20 subjects participated in this study.

### 2.2 Methods

#### 2.2.1 Interview outline confirmation

According to the purpose of the research, through literature search and discussion of the research group, the initial outline of the interview was formulated. An initial round with three nurses helped refine the interview structure, ensuring adaptability until data saturation was reached. The revised interview matrix is detailed in **Table 1**. All participants were informed of the study’s nature and provided their written consent. The study adhered to the Consolidated Criteria for Reporting Qualitative Research and credibility metrics (11, 12): (1) Credibility: Inclusion of field notes and audio captures from the interviews, which underwent team analysis to derive themes. Subsequently, participants authenticated the collated data. (2) Transferability: An exhaustive delineation of the research milieu encompassing participant demographics, sampling logic, and procedural specifics. (3) Reliability: External academic scrutiny ensured the study’s protocol adhered to qualitative research tenets. (4) Confirmability: Triangulation was employed to diminish researcher bias. Self-reflection ensured the recognition of potential limitations, guaranteeing the research’s integrity.

**Table 1:** Theme and sub-themes.

Theme	Subthemes
Career Support & Development	- Institutional & Emotional Management
	- Transparent Communication & Feedback
	- Ways to Realize Self-value
Practical Support & Development	- Stable Resource Allocation
	- Streamlined and Standardized Workflow
	- Training & Assessment Strategies
Personal Support & Development	- Salary Appraisal
	- Nursing Adverse Event Management
	- Adaptive Scheduling
	- Pressure Relief Channels & Organizations

We used a descriptive qualitative research design with a semi-structured approach to interviewing based on purposive sampling, it is an effective strategy to acquire data for qualitative research. It helps to explain, understand, and explore research subjects’ opinions, behavior, and experiences to narrow down the area of research that researcher is interested to discover while listening to them being involved through dialogue.

The research team members were registered nurses who had participated in training related to qualitative research. The finalized interview outline consisted of the following questions: 1) What kind of hospital workplace environment do you expect or desire? 2) Are there any current aspects of your workplace environment that you would like to change? 3) What kind of support do you hope the hospital would provide to help you work better? 4) When faced with adversity, setbacks, trauma, or other negative events at work, what attitude and actions do you expect the hospital to maintain or take? 5) What kind of assistance or support do you expect from the hospital in terms of your personal development? The interview outline was used as a reference, and the interview questions were adjusted according to the actual situation in the interview process to ensure in-depth understanding of the experience of nurses and perspectives on workplace environment needs associated to resilience.

#### 2.2.2 Data collection

A Face-to-face, one-to-one and semi-structured interviews were used to collect data. Semi-structured interview is a research method of in-depth communication that aims to understand the experiences, attitudes and perspectives of research subjects through open-ended questions and flexible feedback mechanisms (13). This research method has also been used in the past to understand the perceptions and perspectives of nurses (14, 15). The researcher acted as interviewer, and arranged time and place for the interview with the participants. The interview took place on the 7th floor of the inpatient Department of the Third People’s Hospital of Henan Province From November 10th to 20th. Each participant was interviewed for 30 minutes after work. Firstly,the researcher introduced herself to the participants before the interview and recorded the location and time of the interview. Secondly, before the interview, the interviewer introduced the informed consent to the participants and asked them to sign their names and dates on the informed consent form. Then, the interviewer asked questions according to the interview outline, and adjusts the questioning strategy flexibly according to the feedback of the participants. Finally, after the interview, the interviewer expressed her gratitude to the participants.

### 2.3 Data analysis

Used the Colaizzi qualitative analysis procedure in this study (16). The interview data were analyzed according to the previously reported steps (17) in **Table 2**.

**Table 2:** Steps in Colaizzi’s qualitative analysis.

Steps	Procedure
1	Assigned an initial code to each nurse. Deeply reviewed each nurse’s interview notes to understand their experiences related to sobriety and their needs concerning psychological resilience.
2	Enhanced dialogue comprehension by refining, identifying, and extracting pivotal statements from interviews, then deducing their meanings.
3	Synthesized and categorized the meaning of pivotal statements into themes, subthemes, and detailed datasets through team collaboration and analysis.
4	Continued interviews until data saturation was achieved, concluding when no fresh topics surfaced.
5	Engaged in discussions to outline the core structure of the nurses’ perceptions and experiences about psychological resilience management.
6	Forwarded the refined text data to participating nurses for validation.

### 2.4 Ethics approval and consent to participate

This study followed the Declaration of Helsinki (18) and was approved by Medical Ethics Committee of The Third People’s Hospital of Henan Province, ethics number: 2023-SZSYKY-023. All participants were informed of the purpose of the study, and all patients participated voluntarily.

## 3 Result

### 3.1 Demographic characteristics of participants

All participants were female, with an average age of 34.55 years (standard deviation of 7.67 years, ranging from 25 to 55 years old). We collected demographic characteristics and baseline data of the nurses, including age, gender, educational level, marital status, monthly income, and number of children. We conducted 25 semi-structured interviews. Interviews and recordings were carried out with the informed consent of the subjects, and the content was transcribed verbatim within 24 hours after each interview. The detailed demographic characteristics of all participants are presented in **Table 3**.

**Table 3:** Demographic characteristics (*n* = 20).

Demographic characteristics	n (%)	Demographic characteristics	n (%)
Age		Children	
<30	8(40.0%)	There are	16(80.0%)
31~40	8(40.0%)	None	4 (20.0%)
41 ~ 50	3(15.0%)	Working Department	
51 ~ 60	1 (5.0%)	Physical examination center	1 (5.0%)
Sex		Intensive Care Unit	2(10.0%)
Women	20 (100%)	Department of gerontology	1 (5.0%)
Men	0 (0)	Department of orthopaedics	1 (5.0%)
Education level		Gynecology and obstetrics	1 (5.0%)
Junior college	9 (45.0%)	Radiology department	1 (5.0%)
Bachelor degree	10 (50.0%)	Disinfection supply center	1 (5.0%)
Master degree or above	1 (5.0%)	Blood purification department	1 (5.0%)
Marital status		Emergency department	2(10.0%)
Married	14 (70.0%)	Department of pediatrics	1 (5.0%)
Unmarried	3 (15.0%)	Pneumology department	2(10.0%)
Divorce	2 (10.0%)	Department of neurology	1 (5.0%)
bereaved spouse	1 (5.0%)	Gastroenterology department	1 (5.0%)
Monthly income		Rehabilitation department	1 (5.0%)
1,000 ~ 4,999	14 (70.0%)	Cardiac surgery	1 (5.0%)
5,000 ~ 9,999	5 (25.0%)	Operating room	1 (5.0%)
>10,000	1 (5.0%)	Traditional Chinese medicine department	1 (5.0%)

### 3.2 Theme 1: Career support and development

#### 3.2.1 Institutional and emotional management

During the interviews, 80.00% of the nurses mentioned their demands with humanized leadership and management styles. These nurses stated that it was important for them to have genuine care and understanding from the head nurse, and to be able to provide them with help and guidance whenever they encountered difficult or uncertain situations in their work, which became a great source of psychological support and provided them with happiness.

“The head nurse always pays attention to our emotional changes and confusions after a day’s work, then takes the time to chat with us like a friend and helps us resolve our doubts.” (N5)

“The nursing department and each ward formulate three measures every year based on nurses’ needs to enhance nurse satisfaction, truly balancing the sleep and training needs of night shift nurses.” (N7)

“Whenever there’s a challenging situation in the ward, our head nurse takes the initiative to bring the team together, not just to find solutions but also to check in on how we’re coping emotionally. It feels like a community where our feelings and well-being matter.” (N18)

Additionally, 30.00% of the nurses mentioned in the interview expressed their need for humanized system management. More humane rules and regulations cannot only increase work efficiency, but also improve the willingness of nurses to abide by the rules. The needs of nurses should be considered in the introduction of relevant management policies to meet the needs of multiple roles and identities of nurses.

“It’s crucial for leadership to remember that behind every nurse, there’s a person with feelings, hopes, and challenges. Systems and processes designed with empathy can truly make a difference in our daily work lives.” (N1)

“I believe that rules and regulations can be both efficient and compassionate. When our human needs are taken into account, we not only follow them more willingly but also take pride in our work and the organization.” (N4)

“If leaders could consider our needs when formulating certain rules, regulations, or requirements and integrate these rules with our needs, I think everyone would implement the relevant rules and regulations more effectively.” (N20)

#### 3.2.2 Transparent communication & feedback

It is important for primary clinical nurses to provide a channel for nurses to feedback and communicate with leaders in time.

In the past, providing feedback was a hassle. Now, the nursing department has established a feedback email for us, and they also hold an annual Nurses’ Representative Conference, giving us opportunities to speak our minds freely.” (N11)

In addition, the establishment of effective and equal communication channels is conducive to the more forceful execution of nursing tasks.

“When the head nurse communicates with me on an equal footing, it motivates me more than simply giving direct orders.” (N9)

“When there are issues with the information system, the bedside Pad is broken, or there are communication problems with delivery or logistics personnel, nurses can contact the relevant department to solve the issue immediately. It’s quite convenient and greatly facilitates our work.” (N20)

Therefore, effective, two-way, and equal communication can also enhance nurses’ sense of ownership and security in their work, allowing them to face emergencies in their work more proactively.

#### 3.2.3 Ways to realize self-value

Realizing one’s self-worth is a reflection of a nurse’s professional career success. It can help nurses obtain positive psychological experiences and a sense of accomplishment, thereby enhancing their psychological resilience. In the interviews, 90% of the nurses mentioned that a true magnet hospital should provide means and methods for nurses to realize their self-worth. Their demands included professional title promotion, becoming a professional nurse and improving their ability. Specific measures could be a clear promotion system, providing practical research capability training, reasonable training and utilization of specialized nurses, and opening nursing clinics for self-realization.

Clinical nurses face difficulties in conducting research and cannot write research papers. They have not received systematic training in school. On the other hand, research papers are still one of the indicators for promotion, so this puts most nurses in a dilemma.

“Research is quite challenging for clinical nurses. Publishing articles is difficult. I hope that in addition to theoretical courses, they can provide some genuine research practice training sessions.” (N6)

“Providing research and training opportunities shows that the management values our contribution and sees the potential in us. This recognition is crucial for our morale.” (N14)

Nurses generally have the demand for continuing education, which involves career planning, nursing education, specialist nurse training and so on. Nurses believed that the special courses arranged by the hospital could make them feel valued, which also made them see the hope of nursing professional development, which could greatly motivate them and make them more active in work.

“Having clear guidelines for promotions, specialized training programs, and opportunities for specialized roles not only make me feel valued but also push me to do my best every day.” (N16)

“In our hospital’s 258 Nursing Plan, I saw the future prospects of the nursing profession. I am fond of nursing education, and I am determined to become a qualified health education nurse.” (N18)

The establishment of nursing clinic can make nurses feel the importance of their specialty, improve their professional identity, and realize that the knowledge they learn has practical value.

“Operating a nursing outpatient clinic enhances our professional identity as nurses. When a patient says ‘thank you’ to me, I feel that my profession is valuable and recognized.” (N19)

“In our hospital, they have opened clinics managed by nurses. This not only allows us to apply our knowledge practically but also provides a sense of achievement.” (N20)

This theme emphasizes the importance of institutional support that values and recognizes the contributions of nurses. By providing them with avenues to grow professionally and realize their full potential, the hospital not only retains its staff but also ensures that they remain motivated and committed.

### 3.3 Theme 2: Practical support and development

#### 3.3.1 Stable resource allocation

The allocation of human and material resources is closely related to the workload and stress levels of nurses. This directly influences the quality and efficiency of nursing services. An irrational structural arrangement can lead to unexpected incidents or accidents in nursing, further reducing the mental resilience of nurses. The interviewed nurses all mentioned the real problems faced in terms of nursing human resources.

There is insufficient staffing in clinical nursing work, which increases the work pressure of nurses and the contradiction between nurses and patients.

“Clinical work is quite busy, with a high intensity and fast pace. The pressure is relatively significant. I hope more nurses can be allocated.” (N3)

“When there are fewer nurses, the time allocated for each patient becomes very limited. It’s easy for patients to have complaints about the nursing work.” (N5)

The application of new technology, the refinement of division of labor, reasonable layout and optimization of resource allocation can reduce the excessive workload of nurses to a certain extent, so as to reduce the work pressure of nurses.

“The reasonable allocation of material resources, such as the emergence of the Internet of Things and informatization, has to some extent made up for the shortage of clinical nursing human resources, alleviating the work pressure of nurses.” (N8)

“Nurses have a large workload and need to juggle multiple tasks simultaneously. The pressure feels immense. Thankfully, the hospital has arranged delivery personnel and implemented pneumatic logistics, so nurses no longer need to run errands outside.” (N9)

“Optimize the shift scheduling system to give our colleagues adequate rest time. When there’s a shortage of staff, we need replacements. We are humans, not machines. Overtime shouldn’t become the norm. Increasing shift personnel is essential, as having eight night shifts in a month is too frequent.” (N12)

This theme highlights the importance of a supportive workplace environment in the nursing profession. By addressing the actual needs and challenges faced by nurses, healthcare institutions can enhance patient care quality and improve nurses’ job satisfaction and overall well-being.

#### 3.3.2 Streamlined and standardized workflow

The demanding nature of nursing roles necessitates a coherent and streamlined workflow. A significant 55.00% of nurses emphasized the need to refine and standardize operations. By doing so, nurses can dedicate more attention to patient care, elevate service standards, and minimize the inherent stresses of their profession.

“We should push toward integrating technology and emphasizing specialized tasks in our workflow. Such an approach not only lightens our workload but also underscores the expertise inherent in nursing.” (N8)

“I hope clinical handover procedures and checklists can be standardized across departments, rather than each department creating their own, to simplify the workflow.” (N9)

“Current workflows have been optimized significantly compared to when I started working; they’re much more detailed. Every shift has its workflow and tasks, and our processes should evolve with the times.” (N11)

“The workflow should lean toward informatization and specialization. This can both alleviate nurses’ workload and showcase the professionalism of nursing.” (N18)

#### 3.3.3 Training and assessment strategies

While training and evaluation play pivotal roles in sharpening the skills of nurses, it’s crucial to strike a balance in their frequency and approach. Every nurse interviewed highlighted the burden of excessive training and evaluations, especially when these overlap with demanding shifts or are masked as ‘relaxing activities’ but end up adding to their workload.

“Following a night shift, many of us nurses are exhausted. Being subjected to intensive training right afterward can be counterproductive, as its advantages are often overshadowed by fatigue. The hospital’s policy that allows nurses finishing night shifts to forgo training is a considerate move.” (N8)

“Why not exempt those who’ve completed night shifts from training sessions? Recovery sleep post such shifts should be prioritized.” (N9)

“Activities pitched as relaxation can feel burdensome if mandated. Forced participation usually diminishes enthusiasm and genuine engagement.” (N13)

“It’s advisable to give a respite from meetings or training for those coming off night shifts. Allocating a dedicated day of rest can significantly boost nurses’ overall well-being.” (N19)

This theme underscores the significance of crafting a workplace environment that respects nurses’ energy and time. By streamlining processes and recognizing the importance of work-life balance, medical institutions can deliver superior patient care while ensuring their nursing staff feels valued and satisfied.

### 3.4 Theme 3: Personal support and development

#### 3.4.1 Salary appraisal

Salary and benefits were among the primary concerns raised by many nurses in terms of personal support.

“The foundational salary doesn’t align with our designated roles; it’s insufficient and barely meets our family needs.” (N9)

“Compensation is a driving force for our dedication. Nurses’ efforts should resonate with their paychecks. It’s imperative to maintain a balance where both regular and contract nurses receive equitable compensation for their services.” (N14)

“I don’t merely have expectations about the amount but also the respect it signifies. I wish society would recognize and value the nursing profession more.” (N16)

Therefore, the rationality of salary and benefits is a significant environmental factor influencing nurses’ psychological resilience.

#### 3.4.2 Nursing adverse event management

Adverse nursing events can lead to panic, anxiety, and tension among nurses. How nurses respond, and whether they can learn and grow from these experiences, are closely tied to the hospital’s approach to managing and handling these incidents. Among the 20 nurses interviewed, 5 had experienced adverse events.

“Actually, no nurse wishes to experience an adverse event. I hope that management can have a standardized and protective procedure for handling these events. It’s vital to offer necessary support for nurses, who are often the second victims in such situations. This support can help alleviate their stress, overcome negative emotions, and return to work more swiftly.” (N4)

“My primary source of stress in my current job stems from the fear that a slight oversight could lead to an adverse event. If such an incident occurs, it affects me both mentally and physically. The gossip among colleagues can be especially distressing.” (N12)

“During my training, they always emphasized accuracy and attention to detail, but no one prepared us for the emotional toll of an adverse event. It’s more than just the incident itself; it’s the after-effects - the sleepless nights questioning your competence, the glances from colleagues, and the silent whispers. Having a supportive system that helps us through these moments is crucial.” (N13)

“I remember the first time I made an error in medication administration. I felt like the weight of the world was on my shoulders. What helped me through it was not only a comprehensive review of the incident but also counseling sessions and the support of my team. We need a safe space to discuss these matters, learn from them, and move on.” (N16)

“In a profession where even the smallest mistakes can have grave consequences, the pressure is immense. I believe it’s not just about how the hospital handles the event but also about creating an environment where nurses can openly discuss errors without fear of retribution. Continuous training and open forums are essential to break the stigma around these events and to ensure they’re minimized in the future.” (N18)

This theme highlights the importance of an institutional response that is both supportive and constructive when dealing with adverse nursing events. It recognizes that nurses, while responsible for patient care, are also susceptible to human errors and should be provided with the necessary resources and support to navigate through these challenging experiences. A culture that prioritizes learning and growth from mistakes, rather than punishment, can foster a more resilient and efficient nursing staff.

#### 3.4.3 Adaptive scheduling

The term “flexible and adaptive scheduling” appeared 25 times in the interview content, indicating that the ability to have flexible scheduling and balance work and life can impact nurses’ psychological resilience.

“Since my family is from out of town, every time before a holiday, the head nurse would ask if I’m going home. If I am, she would arrange the schedule in advance, allowing me to spend an extra couple of days with my family.” (N2)

“Our department is quite considerate when it comes to scheduling. If we have personal matters to attend to and inform the head nurse ahead of time, she tends to be understanding and arranges appropriate shifts.” (N10)

“As long as it doesn’t disrupt the department’s work arrangements, the head nurse seeks input from the nursing staff, and then we can autonomously decide on our annual leave dates.” (N15)

Properly scheduling breaks and vacations can also help alleviate nurses’ mental stress, enabling them to approach work with a more positive mindset.

#### 3.4.4 Pressure relief channels and organizations

Nurses, facing numerous pressures and challenges in their work, need appropriate channels or organizations to release and alleviate stress, establishing a positive emotional approach to deal with challenges or unexpected events.

“While improving patient experience and satisfaction, it’s equally crucial to pay attention to the physical and psychological well-being of the nursing staff, ensuring that their rights and safety are protected. I suggest organizing more group activities and entertainment events to relieve nurses’ stress.” (N7)

“The union’s activities, like the spring and autumn outings we had before, make me feel relatively happier because I can leave my work concerns behind during those times.” (N13)

“The hospital has a Nursing Humanities Committee. I’m actually quite interested in it, but I’m not sure what I would be doing there, and I worry about whether the things I share would be kept confidential.” (N17)

“Conducting regular psychological health assessments and providing nurses with a confidential avenue to express their feelings is essential.” (N18)

Thus, when management offers necessary support to nurses, they should also consider establishing a system to protect nurses’ privacy, allowing them to share their feelings without reservations.

## 4 Discussion

Our interviews found that nurses suggested that the hospital leadership need to develop humanized management, pay attention to nurses’ emotions and confusion, and get along with employees like friends, nurses also believe that hospitals should respect and recognize their contributions, provide professional training opportunities and clear promotion systems, and help nurses realize their professional value. Nurses suggest avoiding excessive training and provide an appropriate scheduling, especially for nurses who have completed night shifts, advocating a balance between work and rest for reducing nurses’ workload. In addition, nurses hope that management can take measures to alleviate their work pressure, such as group building activities, Nursing Humanities Committee, psychological health assessments. Therefore, establishing a humanized management system is an important strategic measure, the new humanizing policy brings a new meaning to the healthcare professional/patient relationship, triggering significant benefits for individuals, groups and organizations (19). Create a positive workplace environment had the power to improve employee performance (20). Similarly, a positive workplace environment also improved the employee commitment level and achievement-striving ability significantly.

During this interview,we found that nurses believe that transparent communication and information feedback are crucial in hospital work, and equal communication and quick feedback help nurses complete their work more effectively, an organization’s day-to-day transparent communication practice characterized by information substantiality, accountability and employee participation largely contributes to employees’ positive evaluation of the organization (21).

Furthermore, nurses want to change the status quo, their salaries are too low to afford family expenses, and their salaries should match the intensity of their work. Optimize the rational allocation of human, material, and financial information resources (22). The low salary level is an important factor affecting the work pressure and job satisfaction of nurses. Research shows that the salary level of nurses in China is relatively low, resulting in the inability to reflect the work value of nurses.

Also, after most nurses experience nursing adverse events, they will be questioned, criticized, and punished, they hope that they can receive support from management. Many nurses express great fear and anxiety regarding the handling of nursing adverse events. Significant negative correlations were found between adverse events and both family and manager support (23). Hospital managers should provide effective support to nurses, including the provision of information and collegial support after adverse events occur.

Based on nurses’ real experiences and the HSWERM model, this study offers a profound understanding of nurses’ support needs regarding psychological resilience in their workplace settings. provides a direction for managers to improve the nursing workplace environment from multiple perspectives and levels, and enhance the psychological resilience of nurses.

### 4.1 Magnetic work policies and structures encourage nurses to provide feedback, promote nurse professional development, and foster lifelong learning

Nursing is unique and complex, and compassion is a moral and spiritual empowerment inherent in nursing. Compassion is applied when the nurse interacts with the patient and shares the pain reflected in their behaviors and attitudes. When nurses perceive and are chronically exposed to nursing stress on the job, it can lead to a state of emotional, physical, and psychological exhaustion. This emotional exhaustion reduces job satisfaction, poorer judgment and discrimination (24). It also reduces the quality of patient care and impairs the nurse-patient relationship. The stressful and high-risk nature of nursing makes the profession more susceptible to symptoms of compassion fatigue than other healthcare professionals (25).On a societal level, it has a negative impact on health care organizations. In addition, in China, the nurse-patient ratio is low. There is a “doctor over care” phenomenon among both patients and health care providers. which creates excessive workloads and emotional stress for caregivers. Under high pressure, it is difficult to provide comprehensive physical and emotional care to every patient. Such adverse feedback can add to the emotional burden of nurses.

Such adverse feedback can add to the emotional burden of nurses. Research has shown that Continuing Professional Development (CPD) in nursing is an important part of the nursing workplace environment. Experienced nurses experience contextual barriers related to lack of support structures and lack of access to CPD resources, lack of support from managers and other colleagues, Lack of avenues for self-worth. Lack of access to CPD resources and activities affects the quality of care and adversely affects nurse satisfaction, recruitment and retention, and reduces nurses’ psychological resilience (26).

This study found that enhancing the support of care managers and care teams was beneficial in increasing caregivers’ sense of belonging, decreasing emotional isolation, and alleviating dysphoria. Expanding different career paths for nurses, exploring possibilities for continued professional development, assisting nurses in realizing their self-worth, achieving a reduction in the risk of damage from a single pathway to fulfillment, improving emotional regulation, optimizing the workplace environment, and increasing psychological resilience.

Effective system management and emotional support significantly impact nurses’ stress perception and coping mechanisms. Establishing transparent and bidirectional communication channels fosters internal motivation within the organization, promoting nurses’ enthusiasm and minimizing negative emotions.

Genetic work policies and structures should focus on two key aspects: encouraging open feedback and nurturing the professional development of nurses. On a broader scale, the implementation of policies centered around human well-being can significantly decrease nurses’ perceived stress (27). Such policies not only enhance their resilience to unforeseen challenges but also contribute to the stability of the nursing team, paving the way for a magnetic hospital environment (28). Crucially, fostering robust, bidirectional communication channels—both within nursing and across disciplines—is essential (29). Effective interprofessional communication ensures that nurses not only receive the respect they deserve but can also anticipate and manage emergencies better. Recent studies, like Bender et al.’s, highlight the potential of innovative interventions like healthcare improvisation communication workshops in improving such communication (30). Moreover, supporting nurses’ professional growth bolsters their psychological resilience. Managers can achieve this by implementing transparent management competitions, streamlined management structures, advanced training modules, and specialized nursing platforms (31). This not only clarifies nurses’ career trajectories but also reinforces their sense of purpose, leading to higher job satisfaction, a positive work cycle, and enhanced psychological resilience (32).

### 4.2 Refining work procedures and adjusting training intensity boost nurses’ care skills

China is a large country with a large population. It has huge medical needs. There are varying degrees of equity in the clustering of the number of RNs in different cities and regions. There is a regional allocation of nursing staff resources, and the overall nurse-patient ratio is substandard. Much evidence suggests that hospital nurse staffing is associated with patient prognosis, and it is also associated with nurses’ mental health (33).Frequent shifts and night shifts can fill a nurse’s life when the nurse-to-patient ratio is out of balance. Complex workflows can add to the workload and result in significant overtime. Excessive operational training and assessment further compresses nurses’ rest time, preventing them from getting timely and quality relaxation. Such a poor workplace environment makes nurses prone to doubt the meaning of life. It makes them suffer from severe anxiety and depression, which damages the mental health of nurses. Reasonable patient-care ratios, adequate supplies, and smoother workflow will optimize the nursing workplace environment. These can reduce unnecessary workloads and enable nurses to focus on patient care, thereby improving the quality of care, reducing nurse burnout, and increasing nurses’ psychological resilience. The study concluded that minimum nurse-patient ratio policies are a viable way to improve nurse staffing and patient outcomes with a high return on investment (34).

Interview findings highlight nurses’ pronounced desire for equitable human resource distribution (35). Sufficient staffing is crucial in alleviating nurses’ workload and stress. In situations with limited staffing, managers are advised to adopt flexible staffing strategies, adjusted for the varying demands throughout different periods and units (36). Embracing streamlined, standardized, and modern work procedures, supplemented by advancements in nursing technology, can further mitigate staffing shortages (37). As delineated in the interviews, innovations like enhanced Personal Digital Assistants (PDAs) and the incorporation of pneumatic logistics significantly diminish nurses’ physical demands (38).

While ongoing training fortifies nurses’ skillsets, it can inadvertently heighten their educational burden. Hence, management should develop talent nurturing models congruent with nurses’ real-world developmental needs (39). By judiciously calibrating training intervals and methodologies, managers can optimize the learning process, ensuring nurses are adeptly prepared for clinical scenarios.

### 4.3 A just, compassionate, and secure work setting offers nurses personal support and room for self-adaptation

Nurses, as an important part of the health care workforce, are paid far less than physicians. Many nurses find it difficult to cope with the enormous pressure of financial life, resulting in an imbalance in the ratio of health care workers to nurses. Disproportionate ratio of medical to nursing, on the one hand, will lead to part of the medical work due to the lack of nursing staff cannot be carried out normally, affecting the physician’s motivation; on the other hand, it will increase the workload of the existing nursing staff, seriously affecting their physical and mental health. A multi-site patient-level longitudinal survey explored the relationship between nurse staffing methods and risk of adverse events (40). Uncoordinated nursing staffing ratios can have an impact on adverse events. Adverse events can have a wide-ranging negative impact on nurse burnout, which in turn affects nurses’ psychological resilience. Adverse events are positively associated with nurse burnout. Adverse events have a greater impact on nurse burnout when nurses strongly identify with their work team, while the impact on nurse burnout is diminished when the safety climate is better. That’s why managers need to focus on creating a favorable safety climate (41).

Salary and associated benefits are paramount determinants of a nurse’s job satisfaction, their experience of occupational stress, and their overall engagement in their roles (42). Our qualitative interviews revealed a consistent theme: nurses, particularly those on contract terms, emphasized the significance of transparent and equitable compensation systems. Advocating for and implementing such systems, characterized by the principle of equal pay for equivalent work, not only supports individual job satisfaction but also underpins the broader stability of psychological resilience within the nursing community (43).

The optimal scheduling of shifts is an intricate balance of addressing the hospital’s operational needs while ensuring nurses have sufficient rest. Beyond just the operational implications, thoughtful scheduling that ensures nurses receive adequate sleep, and has allowances for their personal and family commitments, sets the foundation for sustainable, long-term nursing practices. Such practices translate to reduced burnout and improved patient care (44).

Every medical profession, nursing included, faces the risk of adverse events. Such events can be detrimental, not just from a clinical perspective, but they also carry significant emotional consequences, particularly when amplified by public reactions (45). It’s imperative for institutions to develop a two-fold approach: proactive strategies that aim to minimize such events and reactive strategies that provide support and tools for recovery after such events. Additionally, offering specialized courses that focus on emotional and stress management can offer nurses tools and strategies to navigate these challenging situations, bolstering their psychological resilience (46).

By utilizing the HSWERM model, this research offers a unique lens through which to view and analyze the lived experiences of nurses, particularly concerning their psychological resilience within current practice settings. The findings, which meld both practical implications and theoretical contributions, serve as a foundational guide for the development of tailored interventions aimed at enhancing the workplace environment for nurses. However, it’s crucial to acknowledge the study’s geographical limitation; being concentrated solely on a tertiary general hospital in Zhengzhou might not capture the broader nuances of the nursing community. Therefore, future research endeavors should consider a more expansive, multi-centered approach, incorporating both quantitative and qualitative methodologies, to provide a richer, more comprehensive understanding.

## 5 Limitations

The limitations of this study are mainly related to the study design and small sample size of the qualitative study. In this study, the nurses we interviewed all came from the same hospital, which limited the universality of these nurses’ perspectives among all nurses. Because nurses in other hospitals may hold controversial points because of their different systems and workplace environments.

## 6 Conclusion

The perspectives of nurses for a workplace environment demands needs to be appreciated, and in addition, it is worth noting that the key role of building a good workplace environment in strengthening the resilience of nurses emphasizes the need for careful consideration. Nursing administrators should formulate policies and measures from multiple perspectives based on the real needs of nurses in terms of professional, practical, and personal dimensions.

## 7 Recommendations for future research

To enhance the depth and breadth of understanding concerning environmental factors affecting nurses’ psychological resilience, future investigations should incorporate diverse research methods across varied clinical settings. Such an approach would not only validate the findings of this study but would also offer a repository of actionable insights for nursing managers, policy-makers, and educational institutions. Future research can start from the research design of quantitative research to explore the extent to which the resilience related factors based on the workplace environment affect the resilience of nurses. Based on this, further intervention research can be carried out to explore effective measures to improve the resilience of nurses and improve the workplace environment of nurses. Longitudinal dynamic changes in nurses’ resilience based on relevant workplace environment factors can also be carried out.

## Data availability statement

The original contributions presented in the study are included in the article/Supplementary Material. Further inquiries can be directed to the corresponding authors.

## Ethics statement

The studies involving human participants were reviewed and approved by Medical Ethics Committee of The Third People’s Hospital of Henan Province. The patients/participants provided their written informed consent to participate in this study.

## Author contributions

ML: Conceptualization, Investigation, Methodology, Writing – original draft. RZ: Conceptualization, Investigation, Project administration, Writing – original draft. JW: Data curation, Investigation, Writing – original draft. LZ: Validation, Writing – review & editing. SY: Validation, Writing – review & editing. YT: Visualization, Writing – review & editing. LW: Writing – review & editing. WZ: Resources, Writing – review & editing. XX: Supervision, Writing – review & editing. CH: Conceptualization, Writing – review & editing. ZP: Methodology, Writing – review & editing. RS: Resources, Writing – review & editing.
